# Dexamethasone Regulates CFTR Expression in Calu-3 Cells with the Involvement of Chaperones HSP70 and HSP90

**DOI:** 10.1371/journal.pone.0047405

**Published:** 2012-12-13

**Authors:** Luiz Felipe M. Prota, Liudmila Cebotaru, Jie Cheng, Jerry Wright, Neeraj Vij, Marcelo M. Morales, William B. Guggino

**Affiliations:** 1 Department of Physiology, Johns Hopkins University School of Medicine, Baltimore, Maryland, United States of America; 2 Laboratory of Cellular and Molecular Physiology, Federal University of Rio de Janeiro, Rio de Janeiro, Brazil; 3 Department of Pediatrics and Institute of Nanobiotechnology, Johns Hopkins University School of Medicine, Baltimore, Maryland, United States of America; Cincinnati Children's Hospital Medical Center, United States of America

## Abstract

**Background:**

Dexamethasone is widely used for pulmonary exacerbation in patients with cystic fibrosis, however, not much is known about the effects of glucocorticoids on the wild-type cystic fibrosis channel transmembrane regulator (CFTR). Our aim was to determine the effects of dexamethasone treatment on wild-type CFTR expression.

**Methods and Results:**

Dose–response (1 nM to 10 µM) and time course (3 to 48 h) curves were generated for dexamethasone for mRNA expression in Calu-3 cells using a real-time PCR. Within 24 h, dexamethasone (10 nM) showed a 0.3-fold decrease in CFTR mRNA expression, and a 3.2-fold increase in αENaC mRNA expression compared with control groups. Dexamethasone (10 nM) induced a 1.97-fold increase in the total protein of wild-type CFTR, confirmed by inhibition by mifepristone. To access surface protein expression, biotinylation followed by Western blotting showed that dexamethasone treatment led to a 2.35-fold increase in the amount of CFTR in the cell surface compared with the untreated control groups. Once protein translation was inhibited with cycloheximide, dexamethasone could not increase the amount of CFTR protein. Protein stability was assessed by inhibition of protein synthesis with cycloheximide (50 µg/ml) at different times in cells treated with dexamethasone and in untreated cells. Dexamethasone did not alter the degradation of wild-type CFTR. Assessment of the B band of CFTR within 15 min of metabolic pulse labeling showed a 1.5-fold increase in CFTR protein after treatment with dexamethasone for 24 h. Chaperone 90 (HSP90) binding to CFTR increased 1.55-fold after treatment with dexamethasone for 24 h, whereas chaperone 70 (HSP70) binding decreased 0.30 fold in an immunoprecipitation assay.

**Conclusion:**

Mature wild-type CFTR protein is regulated by dexamethasone post transcription, involving cotranslational mechanisms with HSP90 and HSP70, which enhances maturation and expression of wild-type CFTR.

## Introduction

Cystic fibrosis channel transmembrane regulator (CFTR) is a plasma membrane cyclic AMP-activated chloride channel expressed in several tissues, such as the kidney, pancreas, intestine, vas deferens, sweat ducts, and lungs [1], [2]. It functions to create a thin layer of mucosal fluid in most tissues where it is expressed [3]. In the sweat ducts,it drives salt reabsorption [4]. In kidney, it functions to regulate other channels such as ROMK [5]. The precise regulation of CFTR-mediated chloride transport across epithelial cells such as the lung is important to maintain the proper height of the surface liquid in the airway [6]. In the gastrointestinal tract, proper regulation of the mucosal fluid is important for the passage of food [7]. This is evident on two fronts. In cystic fibrosis, too little fluid causes intestinal obstruction and too much fluid causes diarrhea [8]. Given that CFTR is an ion channel, regulation of its function can occur by the modulation of single channel activity [9]. For example, in cholera an excessive increase in channel activity can cause extreme loss of fluid from the body. Alternatively, modulation of function can occur by regulation of the channel number by altering gene [10], [11] and/or protein expression. Given that CFTR is a plasma membrane protein, the channel number can be affected by insertion and/or removal from the plasma membrane [12] and by modulating total protein levels through alterations in synthesis and degradation [13].

Disruption of CFTR function occurs in cystic fibrosis as a result of genetic mutations [14]. ΔF508 mutation is the most common in Caucasians and causes dysfunction in its channel activity, premature degradation, and failure to traffic to the plasma membrane [15], [16]. Defective CFTR function at the cell surface leads to drastically reduced secretions from airway epithelia, inducing persistent lung infectionsfollowed by fibrosis of lung parenchyma [17], intestinal obstruction, infertility in males, destruction of the pancreatic ducts, and high sweat chloride, symptoms common inpatients with cystic fibrosis [18]. Thus, considerable effort has been made to understand the function and regulation of CFTR and studies have focused on the discovery of molecules that regulate CFTR degradation, processing, trafficking, and activity as noveltargets for drug discovery [19]–[21].

The biosynthesis of CFTR is relatively rapid but the assembly of normal CFTR into a functional channel is not completely efficient and, in some cell types, about 60%–75% of wild-type CFTR is degraded [13]. Current models suggest that the folded state of wild-type CFTR and its most common mutation, ΔF508-CFTR, is monitored by the cytosolic chaperones HSP70 and HSP90 [22]–[24]. Cytosolic HSP70 functions in complexes with co-chaperones and ligases that mediate steps in CFTR folding and degradation [25]. In contrast, interaction of CFTR with HSP90 facilitates channel maturation and folding, helping CFTR to process to the cell membrane [23].

It is known that low doses of dexamethasone slow the progression of lung disease in patients with cystic fibrosis through its role in reducing the inflammation associated with the bacterial infection [26]. However, glucocorticoids may have an additional role in affecting CFTR. In a recent study, glucocorticoids have been shown to rescue ΔF508-CFTR by inhibiting Nedd4-2, a ubiquitin ligase [27]. Inhibition of the ligase reduces ubiquitination of CFTR, ultimately reducing degradation and promoting the processing of ΔF508-CFTR to mature band C. It is well known that Nedd4-2 plays a major role in regulating ENaC surface expression by binding to ENaC via the interaction between ENaC's PP*X*Y motifs and Nedd4-2's WW domains. Nedd4-2 promotes the ubiquitination of ENaC, targeting it for proteasomal degradation [28]. Nedd4-2 is itself regulated through phosphorylation by SGK1. The net effect of phosphorylation is to maintain ENaC at the plasma membrane [28]. The effect of glucocorticoids in rescuing ΔF508-CFTR is also thought to occur via SGK1 inhibition of Nedd4-2 [27]. We noticed in the published report that glucocorticoids also had a dramatic effect on increasing the steady state amounts of wild-type CFTR protein but only had a modest effect on increased membrane retention [27]. This suggests that mechanisms beyond inhibition of degradation of wild-type CFTR were involved in increasing the amounts of steady state wild-type protein. In this present study, dexamethasone does indeed utilize additional pathways to enhance wild-type CFTR expression in human bronchial cells (Calu-3 cells) through a differential effect on HSP70 and HSP90.

## Methods

### Cell line, culture conditions, and reagents

Calu-3 cells (from ATCC, catalog no. HTB-55) were used in the study. The cells were cultured in minimum essential Eagle's medium (MEM, Invitrogen), 10% fetal bovine serum (FBS, Invitrogen), 1% penicillin/streptomycin at 37°C in a humidified incubator in 5% CO2. Prior to each drug treatment, the cells were kept in MEM for 24 h without FBS. Dexamethasone, mifepristone, and spironolactone were acquired from Sigma and diluted in ethanol in stock solutions at 10 mM and stored in the freezer at −20°C. Cycloheximide solution (100 mg/ml, Sigma-Aldrich) was kept at 4°C diluted in dimethyl sulfoxide (DMSO).

### RNA isolation

Total RNA was extracted from the Calu-3 cells grown in six-well plates with or without dexamethasone. First, a dexamethasone dose–response curve was designed using different concentrations (1 nM, 10 nM, 100 nM, 1 µM, and 10 µM) within 24 h, and then a time–response curve was obtained (3, 24, and 48 h) using 10 nM dexamethasone. At the end of each experiment, an RNeasy column kit (Qiagen) was used to purify the RNA, including a one-step treatment with RNase-Free DNase Set (Qiagen). RNA samples were quantified in a spectrophotometer and the ratio of absorbance at 260 nm and 280 nm was used to assess the purity of the RNA. A ratio of ∼2.0 was generally accepted for the samples. The total RNA samples were then used for the microarray technique and/or reverse transcription followed by a quantitative realtime polymerase chain reaction (PCR) (qRT-PCR).

### Quantitative real-time polymerase chain reaction

qRT-PCR was used to test the changes in expression of αENaC, CFTR, and β-actin; microarray analysis was also used. qRT-PCR was performed using total RNA (1 µg) extracted from the cell cultures as described above. Transcripts of the target genes were amplified using gene-specific primers and β-actin was used as the control: CFTR (111 bp) sense 5′-AAG CTG TCA AGC CGT GTT CT-3′and antisense 5′-GAT TAG CCC CAT GAG GAG TG-3′; αENaC (110 bp) sense 5′-TGC TGC GCG CAG AGC AGA ATG-3′ and antisense 5′-TTC CTC ATG CTG ATG GAG GTC-3′: and β-actin (118 bp) sense 5′-TAT TGG GCG CCT GGT CAC CAG G-3′ and antisense 5′-CGG TGC CAT GGA ATT TGC CAT G-3′. Reverse transcription (RT) was performed using a SuperScript III kit (Invitrogen). The cycles for the qRT-PCR were: 15 min at 95°C, 40 cycles of 15 s at 95°C, 30 s at 60°C, and 35 s at 72°C. An Applied Biosystem Thermal Cycler 7500 was used to run the reactions. Sample fluorescence was read at 78°C (3–5°C below the peak melting temperature for each specific cDNA) to exclude contributions from nonspecific sources such as primer dimers. To exclude the possibility of genomic DNA and nonspecific RNA amplification during the qRT-PCR reaction, no-template controls were performed and accepted when the threshold value (Ct) was at least nine cycles greater than the template run. Measurements were performed in duplicate and accepted if the difference between the Ct values of the duplicates was less than 1. The generation of a single product of appropriate size was routinely checked by the presence of a single melt peak and by agarose gel electrophoresis. Data were analyzed using 7500 System SDS software. A relative expression method was implemented, normalizing the data by the internal control β-actin and expressing the final result as a percentage of the control group.

### Immunoblotting

Cells were harvested and solubilized in lysis buffer (50 mM Tris–HCl, pH 7.4, 150 mM NaCl, 1 mM ethylenediamine tetraacetic acid (EDTA), 2 mM EGTA, 1.0% Nonidet P-40) and complete protease inhibitor (Roche Diagnostics, Mannheim, Germany). The cell lysates were centrifuged at 14,000×*g* for 15 min at 4°C to obtain pellet-insoluble material. The supernatants were subjected to SDS-PAGE and Western blotting followed by enhanced chemiluminescence (Amersham Biosciences). The chemiluminescence signal on thepolyvinylidene difluoride membrane was directly captured by a FujiFilm LAS-1000 plus system with a cooled CCD camera. Quantification was carried out within the linear range using Scion Image 4.0 software. CFTR protein was detected with monoclonal anti-human CFTR (C terminus) antibody (R & D Systems, Inc.); glyceraldehyde-3-phosphate dehydrogenase (GAPDH), used as a loading control, was detected with monoclonal GAPDH antibody (US Biological); and chaperones were identified with HSP90 (Stress Gen) and HSP70 (Stress Gen) monoclonal antibodies. The experimental groups were designed as follows: Ctrl, acontrol group without drug treatment; Dx, group treated with 10 nM dexamethasone; Dx+Mif, group treated with 10 nM dexamethasone plus 1 µM mifepristone (an inhibitor of glucocorticoid receptors); Mif, group treated only with 1 µM mifepristone; Dx+Spir, group treated with 10 nM dexamethasone plus 1 µM spironolactone, an inhibitor of mineralocorticoid receptors; Spir, group treated with 1 µM spironolactone. All groups were treated for 24 h.

### Surface protein biotinylation of CFTR

The cell surface proteins were labeled with cell-impermeable EZ-LinkTM Sulfo- NHS-SS Biotin (sulfosuccinimidyl-2-(biotinamido) ethyl-1,3-dithiopropionate; Pierce) at 4°C for 15 min. The cell surface proteins were isolated from the lysate by incubating with immobilized NeutrAvidin beads at 4°C for 2 h (Pierce; catalog no. 53151). The bound proteins were eluted with 2× Laemmli sample buffer supplemented with 100 mM dithiothreitol at 42°C for 30 min. The eluted proteins were subjected to SDS-PAGE and Western blotting followed by SuperSignal West Dura (Thermo Scientific). The experimental groups were: Ctrl, the control group without drug treatment; and Dx, treated with 10 nM dexamethasone for 24 h.

### Immunoprecipitation

Cells were lysed directly on plates using protein lysis buffer (50 mM Tris–HCl, pH 7.4, 150 mM NaCl, 1 mM ethylenediamine tetraacetic acid (EDTA), 2 mM EGTA, 1.0% Nonidet P 40), and complete protease inhibitor (Roche Diagnostics, Mannheim, Germany) after washing with ice-cold phosphate-buffered saline. Total protein extracts (500 µg/ml) were incubated overnight at 4°C with 2 µg of CFTR 14-1 antibody (R & D Systems). Then, 25 µl of protein A/G-agarose beads (Santa Cruz Biotechnology, Inc.) were added to the lysate and rotated for 3 h at 4°C. Beads were washed three times with lysis buffer and suspended in Laemmli sample buffer (50 µl) containing 5% β- mercaptoethanol. Samples were then incubated at 42°C for 20 min and total protein content protein was measured using a 4–15% polyacrylamide gel (Bio-Rad) subjected to Western blotting for HSP70 or HSP90.

### Metabolic labeling

Thirty minutes before the addition of radiolabeled amino acids, the tissue culture medium was replaced with methionine/cysteine-free minimal essential medium. After 30 min of amino acid starvation at 37°C, 300 µCi/ml *trans*-([35S]methionine/cysteine) was added, and cells were pulse-labeled for 5 and 15 min. Cells were lysed at the time points indicated and CFTR was immunoprecipitated using the 24-1 monoclonal antibody (B&D System) and protein A/G-agarose (Roche Diagnostics). Immunoprecipitated samples were analyzed by SDS-PAGE (5% gels) and detected using autoradiography (PhosphorImager, Amersham Biosciences).

### Statistics

Results were submitted to one-way ANOVA with the Tukey post test, usingOrigin 8 biostatistics software. Values were considered significant at *p*<0.05; all measurements were done at least three times.

## Results

**Figure 1:**
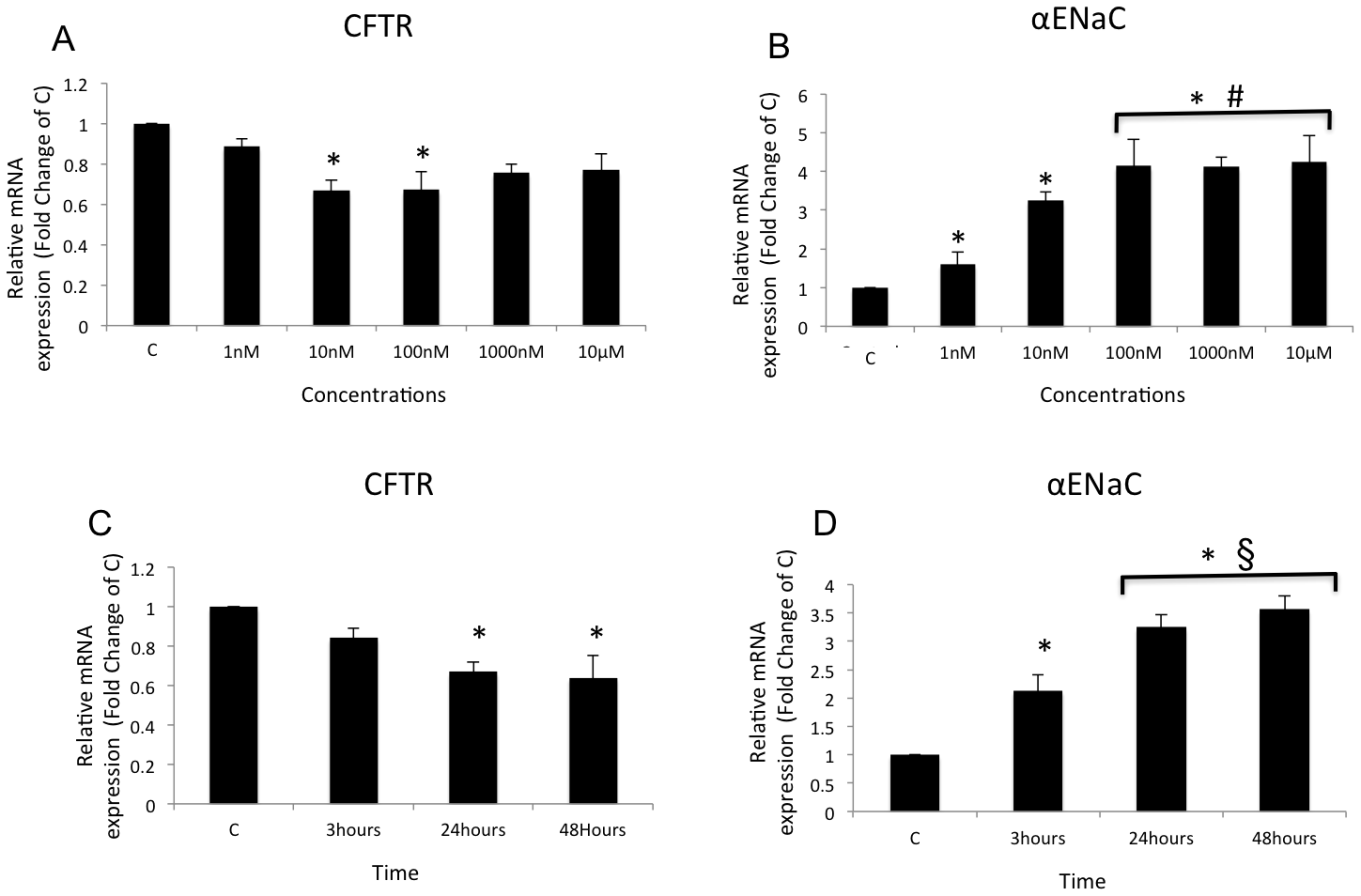
Effect of dexamethasone on CFTR and αENaC mRNA expression. Graphs represent the mean ± SE for mRNA relative expression of CFTR and αENaC acquired by real-time PCR. Dose–response curves for dexamethasone on (**A**) CFTR and (**B**) αENaC mRNA expressions. *Significant difference compared with the control (C) group #significant difference compared with the group treated with 1 nM dexamethasone; *p*<0.05, *n* = 5. Time course of (**C**) CFTR and (**D**) αENaC mRNA expression in response to treatment with 10 nM dexamethasone for 3, 24, and 48 h. *Significant difference compared with the control group; §significant difference compared with the group treated for 3 h; *p*<0.05, *n* = 4.

To examine the effects of dexamethasone on CFTR mRNA expression, cells were treated for 24 h in a cell medium containing different concentrations of dexamethasone (1, 10, 100, 1000 nM, and 10 mM). CFTR mRNA expression decreased by 0.33-fold in cells treated with 10 and 100 nM dexamethasone compared with controls (*n* = 5, *p*<0.05) (Fig. 1A). As a positive control for the effects of dexamethasone, we showed that αENaC mRNA expression was upregulated at all dexamethasone concentrations tested, reaching a plateau of modulation over 100 nM, 1000 nM, and 10 mM (*n* = 5, *p*<0.05; Fig. 1B) consistent with the results from other studies [29], [30]. The time course of the effect was determined at different time points (3, 24, and 48 h) using a single concentration of 10 nM dexamethasone to evaluate changes in mRNA expression of CFTR and αENaC (Fig. 1C and D, respectively). CFTR mRNA expression decreased only in the cells treated with dexamethasone for between 24 and 48 h (0.33- and 0.36-fold, respectively, *n* = 4, *p*<0.05). αENaC mRNA expression was increased after 3 h, 24 h, and 48 h of dexamethasone treatment (2.13-, 3.25-, and 3.57-fold, respectively, *n* = 4, *p*<0.05).

**Figure 2:**
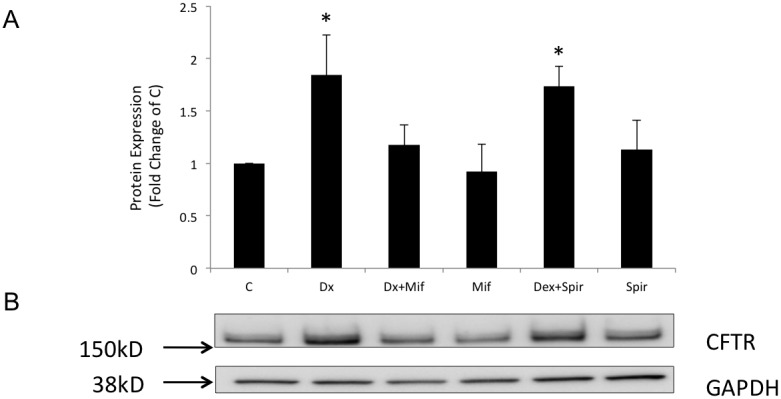
Glucocorticoid and mineralocorticoid receptor inhibition by mifepristone and spironolactone, with or without dexamethasone (Dx) following 24 h of treatment. (**A**) Graphs representing the densitometric values of protein expression from the blots normalized by GAPDH and the respective blots showing CFTR and GAPDH protein expression in the experimental groups (**B**). *p<0.05, *n* = 4.

**Figure 3:**
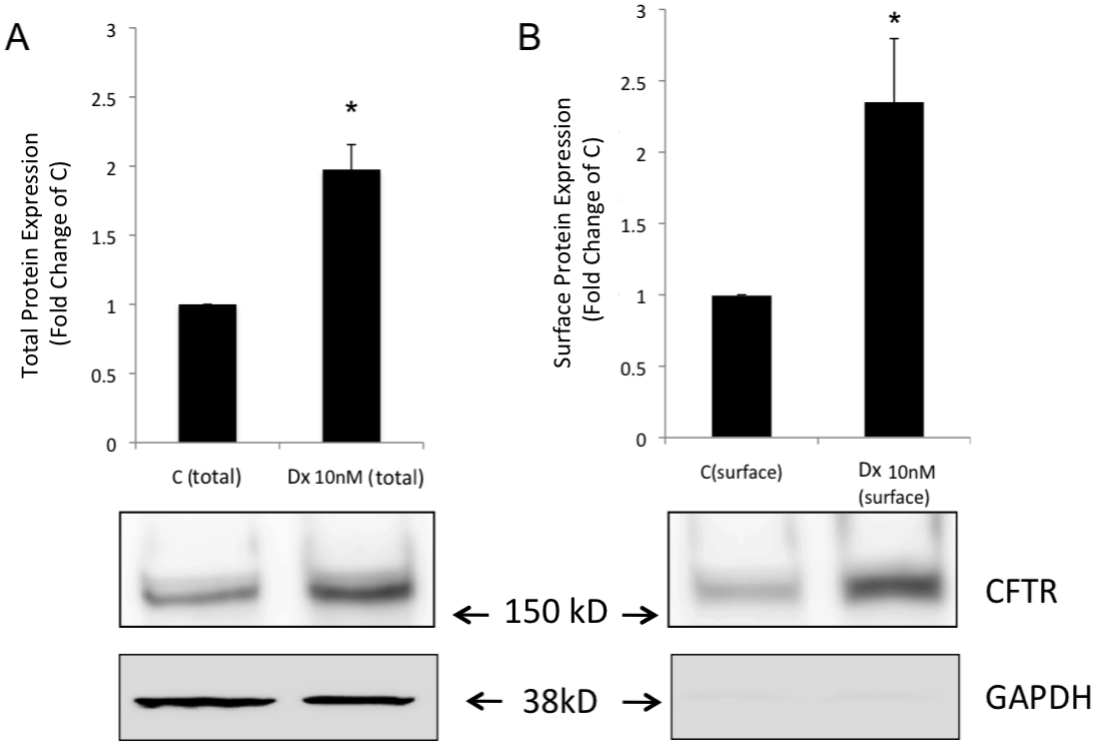
Total protein expression and surface protein biotinylation following treatment with dexamethasone (Dx, 10 nM) 24 h. (**A**) Graphs represent the means ± SE for densitometric values of CFTR total (**A**) and surface (**B**) protein expression and respective blots showing CFTR and GAPDH total and surface protein expression for the control, C (total), and after treatment with dexamethasone (10 nM), Dx (total), for 24 h. *p<0.05, *n* = 3 (pooled with 2 samples per N).

We next determined the effect of dexamethasone on the amounts of CFTR protein. In addition, to pinpoint which steroid receptor was involved in the CFTR effect, dexamethasone was applied to cells in the presence and absence of glucocorticoid and mineralocorticoid receptor inhibitors. Mifepristone (1 µM) and spironolactone (1 µM) were used to block glucocorticoid and mineralocorticoid receptors, respectively, in cells treated with 10 nM dexamethasone. The results summarized in Fig. 2B show that CFTR total protein expression was increased by 1.84-fold (*n* = 4, *p*<0.05) in cells treated with 10 nM dexamethasone for 24 h. This increased expression was inhibited when cellswere treated with dexamethasone plus mifepristone. Spironolactone did not inhibit the stimulation of CFTR expression with dexamethasone (Fig. 2). These data suggest that the dexamethasone effect occurred primarily via the glucocorticoid receptor. Consistent with an increase in CFTR protein, the cell surface biotinylation assay showed that CFTR expression at the cell surface was increased 2.20-fold when cells were treated with dexamethasone, compared with the untreated control group (*n* = 3, *p*<0.05) (Fig. 3B).

**Figure 4:**
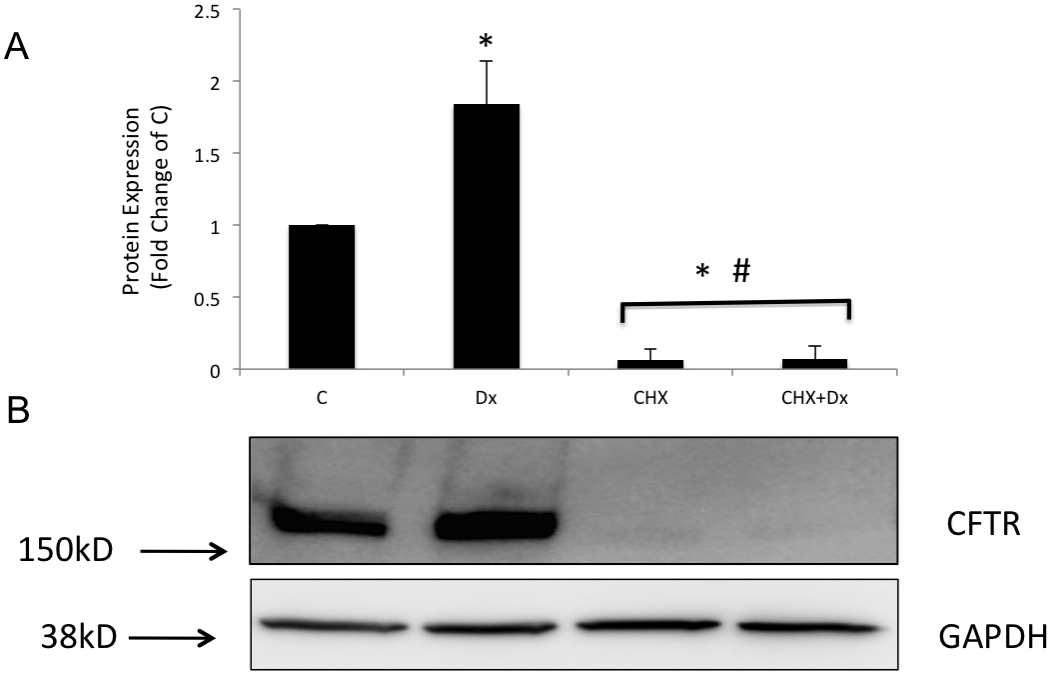
Inhibition of protein synthesis by cycloheximide and treatment with dexamethasone for 24 h in Calu-3 cells. (**A**) Graphs representing means ± SE for densitometric values of respective blots showing CFTR and GAPDH protein expression in C, control group; Dx, dexamethasone 10 nM; CHX, cycloheximide (50 µg/ml) treated group; and CHX+Dx, cycloheximide (50 µg/ml) plus dexamethasone (10 nM) treated group. *Significantly different from the C group; #significantly different from the Dx group; *p*<0.05, *n* = 4.

**Figure 5:**
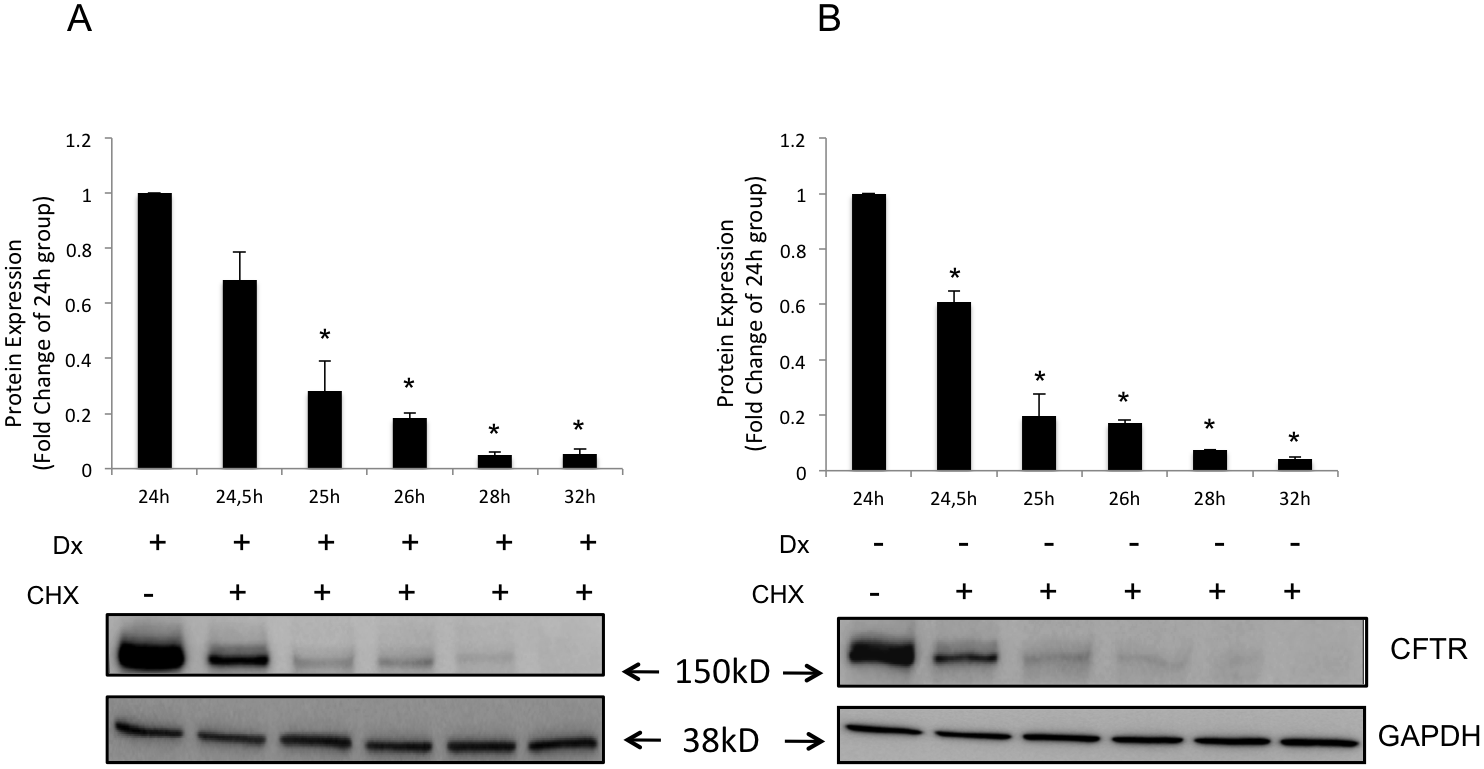
CFTR protein expression in Calu-3 cells in the presence of cycloheximide with or without dexamethasone. Calu-3 cells were treated (**A**) with (+) or (**B**) without (−) 10 nM Dx for 24 h, and then incubated in the presence of 100 µg/ml cycloheximide (CHX) for the indicated times (0.5, 1, 2, 4, and 8 h). In the graphs, CFTR protein expression is represented by the densitometric values of each blot using GAPDH as a housekeeper protein. For CHX+Dx, the protein expression was normalized by the group treated with dexamethasone for 24 h and for CHX the normalization was related to the C group. *Significantly different from the respective 24-h group; *p*<0.05, *n* = 4.

To address whether dexamethasone was reducing the degradation of wild-type CFTR, we inhibited protein translation by treating the cells with cycloheximide (50 µg/ml) for 24 h and found that the total amount of CFTR protein expression (analyzed by Western blotting) was decreased in cells either in the presence or absence of 10 nM dexamethasone (Fig. 4). When cells treated with dexamethasone for 24 h were subsequently treated with cycloheximide (50 µg/ml) for 30 min, 1 h, 2 h, 4 h, and 8 h, 0.32-, 0.72-, 0.82-, 0.96-, and 0.95-fold reductions, respectively, in the amount of CFTR protein were observed compared with the group of cells treated only with dexamethasone for 24 h (Fig. 5A). The addition of cycloheximide (50 µg/ml) to the cell medium for 30 min, 1 h, 2 h, 4 h, and 8 h in cells not subjected to previous dexamethasone treatment showed a decrease in the amount of CFTR protein by 0.40-, 0.81-, 0.83-, 0.93-, and 0.96 fold respectively compared with the control (Fig. 5B). The results showed that dexamethasone did not have a significant effect on the degradation of wild-type CFTR.

**Figure 6:**
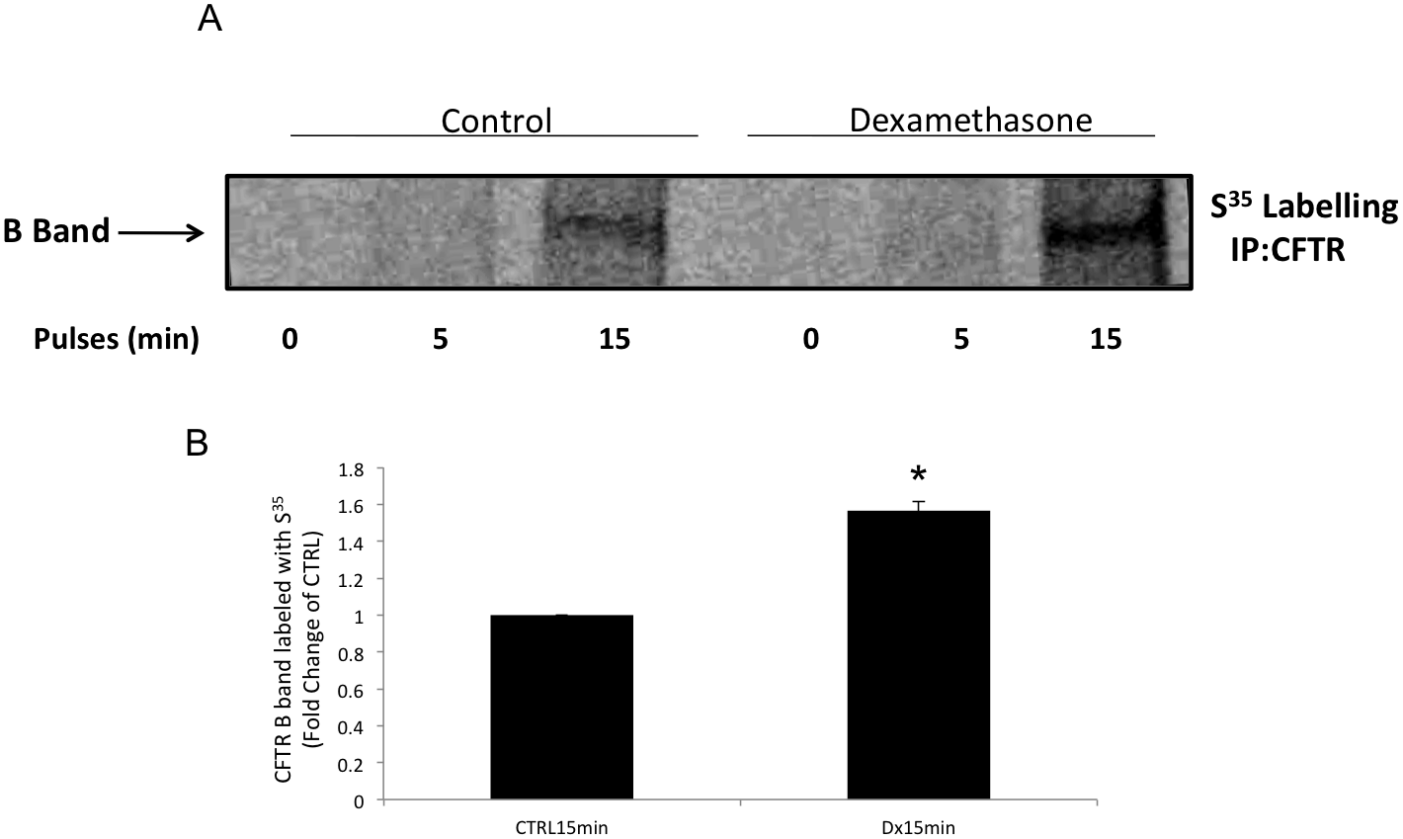
CFTR protein expression in Calu-3 using [35S]methionine pulse-labeling analysis. Pulse labeling with 35S for 0, 5, and 15 min after a 24-h cell treatment with or without 10 nM dexamethasone. (**A**) Blot representing the CFTR band (**B**) protein labeled with 35S on a radiographic film. The graphs represent the densitometric values of the bands shown in the blot for CFTR. *n* = 3, *p<0.05.

**Figure 7:**
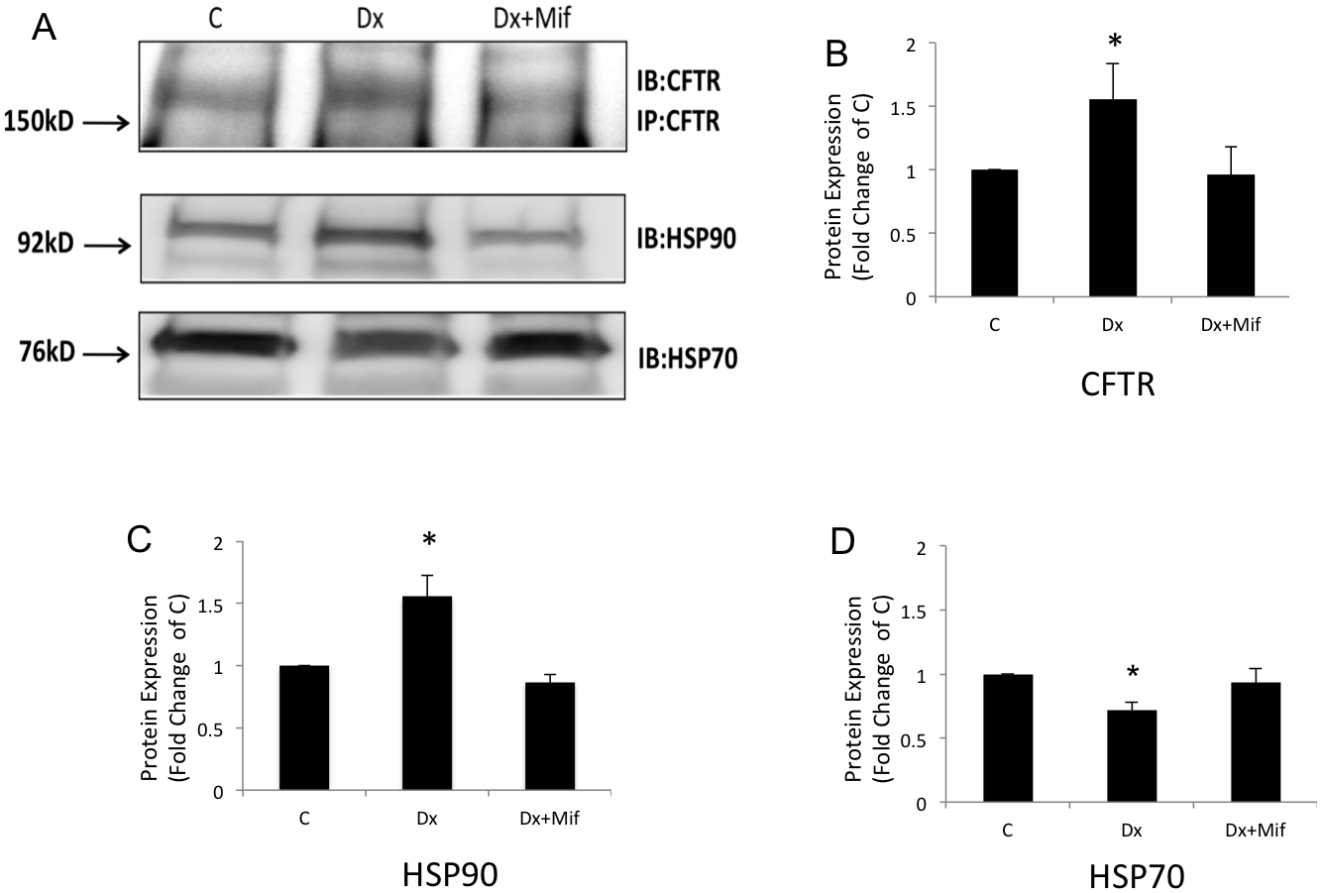
Immunoprecipitation assay for CFTR protein followed by Western blot for HSP90 or HSP70 in Calu-3 cells. Cells were treated with (Dx) or without (C group) dexamethasone or with mifepristone (Dx+Mif) for 24 h and total cell lysate. (**A**) Immunoprecipitation with CFTR Ab and subsequent Western blot for HSP90 or HSP70. The graphs represent the densitometric values of the bands shown in the blots for (**B**) CFTR, (**C**), HSP90, and (**D**) HSP70. Results are presented as the average and SE. *n* = 4, *p*<0.05.

Because dexamethasone did seem to affect wild-type CFTR degradation, we evaluated the early synthesis of CFTR by adding 35S for 0, 5, or 15 min to Calu-3 cells either treated with dexamethasone for 24 h or untreated. This method allowed for the evaluation of early protein synthesis of the B band of CFTR. 35S-labeled CFTR was detected only after 15 min in both groups of cells (treated with dexamethasone or untreated). However, CFTR synthesis was increased by 1.52-fold (*n* = 3, *p*<0.05; Fig. 6B) when cells were subjected to dexamethasone treatment compared with untreated control cells, suggesting that more was indeed synthesized in response to dexamethasone. Because we found that dexamethasone was enhancing the early synthesis of CFTR, we tested whether the molecular chaperones, HSP70 and HSP90, were involved. To accomplish this, an immunoprecipitation assay for CFTR protein followed by Western blotting for HSP90 or HSP70 in the cells treated with dexamethasone or dexamethasone plus glucocorticoid receptor inhibitor (mifepristone) over 24 h was performed. Fig. 7A shows the blots with the bands representing each group described above. As shown above, CFTR protein expression was increased by 1.55-fold (*n* = 4, *p*<0.05) (Fig. 7B) after treatment with 10 nM dexamethasone for 24 h. Interestingly, the amount of HSP90 immunoprecipitated by CFTR increased by 1.55-fold (*n* = 4, *p*<0.05) (Fig. 7C) whereas, within the same time, the amount of HSP70 was decreased by 0.30- fold (*n* = 4, *p*<0.05) in the same cells treated with dexamethasone (Fig. 7D). The addition of mifepristone, a glucocorticoid receptor inhibitor, avoided such modulations (Fig. 7B–D). HSP70 and HSP90 in the total protein lysate studied by Western blotting did not show any difference in expression in cells treated with dexamethasone or dexamethasone plus mifepristone for 24 h compared with the control.

## Discussion

This study was conducted to evaluate the effects of dexamethasone treatment on CFTR expression in a serous cell model, Calu-3 cells (lung adenocarcinoma), and to understand the mechanisms involved in such regulation. The Calu-3 cell line is a very well-studied model for testing bronchial serous cell drug delivery and well characterized to express large amounts of CFTR [31]. We showed that dexamethasone at very low doses (10 nM and 100 nm) depresses CFTR mRNA expression in Calu-3 cells treated for 24 h but not for 3 h (Fig. 1C). Other studies on bronchial cell lines subjected to dexamethasone treatment at a concentration of 100 nM for 24 h have shown that αENaC mRNA is also upregulated in other genes, but not in CFTR [32]. This difference could be explained by differences in the glucocorticoid response elements (GRE) between expression in these cell lines [14]. Similar to the published reports, we found that αENaC was upregulated by the dexamethasone treatment at all doses of the drug [1, 10, 100, 1000 nM, and 10 µM) (Fig. 1B) after 3, 24, and 48 h of treatment (Fig. 1D). Dexamethasone acts directly on the promoter of the αENaC gene by stimulating GRE and increasing mRNA transcription [33].

Despite the suppression of CFTR mRNA expression, the steady state amounts of CFTR protein were upregulated by almost 2-fold, indicating that dexamethasone modulates CFTR protein expression at the post-transcriptional level. Such a large increase in the total amount of mature CFTR could be responsible for the approximately 2.20-fold increase in the amount of CFTR protein induced by dexamethasone that we observed at the cell surface (Fig. 3B). Physiologically, when CFTR is on the cell surface, it functions as a chloride channel and a regulator of the conductance of other channels, such as ENaC. CFTR is an important inhibitor of ENaC function by decreasing its Na+ absorption [34]. Under somephysiological conditions, glucocorticoids may activate both pathways and increase the turnover at the subbronchial gland cell level while maintaining a normal balance between liquid secretion and absorption. The mechanisms involved in such a process require further study.

We have clearly shown that the effect of dexamethasone on CFTR occurs in a specific way via the glucocorticoid receptor (Fig. 2) corroborating the results of Caohuy et al. [27]. They also that upregulation in the mature C band of wild-type CFTR was induced by dexamethasone treatment, similar to our study. We also observed that dexamethasone had little effect on the degradation of the mature band of wild-type CFTR as seen in the cyclohexamide assay (Fig. 5), and thus we surmised that mechanisms other than degradation were involved, which could envolve molecular chaperones.

Several years ago,Yang et al. [35] showed that HSP70 could be immunoprecipitated with CFTR. They showed that HSP70 dissociates from CFTR during movement to the Golgi. In contrast, HSP70 remains associated with ΔF508-CFTR in the endoplasmic reticulum (ER) suggesting that HSP70 plays a role in blocking ΔF508-CFTR transport out of the ER. In subsequent studies, Loo et al. [23] also showed that HSP90 plays a role in the early maturation of CFTR. It is also well known that several chaperones, especially HSP90, affect the functioning of steroid receptors, such as glucocorticoid receptor [36]. In light of these studies, we tested the effect of dexamethasone treatment on HSP70 and HSP90. We have shown that 10 nM dexamethasone treatment over 24 h increased the amount of HSP90 bound to CFTR by 1.55-fold (Fig. 7C) but decreased HSP70 binding by 0.30-fold (Fig. 7D) with no change in the total amount of either chaperone. It seems that dexamethasone alters the balance of HSP70 and HSP90 toward the binding of HSP90. We speculate that, because of this, less CFTR is caught up in the protein quality control process through HSP70, more CFTR moves towards the Golgi complex through HSP90 targeting, and more CFTR is expressed in the cell. This scenario is consistent with the observations presented here.

Our work is significant because it raises the possibility of new strategies to increase CFTR, leading to new therapies for cystic fibrosis in which dexamethasone is likely to change the balance between HSP70 and HSP90 in such a way as to promote maturation of newly synthesized CFTR, as we have shown here.
